# miR-22-Galectin-1 as an integral signaling axis in regulating metabolism and immunity in HCC

**DOI:** 10.1186/s40364-025-00838-3

**Published:** 2026-01-15

**Authors:** Ying Hu, Tahereh Setayesh, Prasant Kumar Jena, Yutong Ji, Trenton Testerman, Ruiwu Liu, Tsung-Chieh Shih, Xiao-Jing Wang, Fu-Tong Liu, Kit S. Lam, Yu-Jui Yvonne Wan

**Affiliations:** 1 Department of Pathology and Laboratory Medicine, University of California Davis, Sacramento, CA USA; 2 Department of Pediatrics, Cedars Sinai Medical Center, Los Angeles, CA USA; 3 Department of Biochemistry and Molecular Medicine, University of California Davis, Sacramento, CA USA; 4 Department of Dermatology, Keck School of Medicine, University of Southern California, Los Angeles, CA USA

**Keywords:** Complement, Immunity, Rho GTPase, Glycan, Liver

## Abstract

**Background:**

While miR-22 is a suppressor of hepatocellular carcinoma (HCC), galectin-1 (Gal-1) serves as a HCC biomarker. Our previous studies have shown the effectiveness of miR-22 gene therapy and silencing Gal-1 as two potential novel options in treating HCC in preclinical mouse models. This study examines the significance of the miR-22-Gal-1 axis in HCC development and treatment.

**Methods:**

The roles of miR-22 and Gal-1 in human HCC were analyzed using the Cancer Genome Atlas database based on their expression levels. The temporal effects of miR-22 were studied by analyzing signaling pathways affected by miR-22 expression levels during HCC progression. AAV8-miR-22, AAV9-Gal-1 siRNA, and LLS30, a Gal-1 inhibitor, were used to treat orthotopic mouse HCC. Spatial transcriptomics established the location-specific effects of miR-22 in mouse HCC. The signaling pathways affected by miR-22 and Gal-1 were identified by analyzing human HCC transcriptomics compared with those found in miR-22, Gal-1 siRNA, or LLS30-treated mouse HCC.

**Results:**

In the early stages of HCC, miR-22-high HCC exhibited extensive upregulation of endobiotic metabolism and xenobiotic detoxification signaling, accompanied by the activation of complement and clotting cascades. In late HCC stages, miR-22-high HCC exhibited heightened innate and adaptive immunity, associated with increased interferon signaling. These impacts were primarily observed in the tumors. At the tumor margin, miR-22 inhibited the Rho GTPase and cell–matrix interaction, revealing its role in reducing matrix remodeling and mobility. In non-tumor areas, miR-22 inhibited inflammation by reducing neutrophil degranulation, platelet activation, chemokine receptor binding, and fiber formation. miR-22, Gal-1 silencing, and LLS30 each exhibited anti-HCC effects and targeted common intracellular signaling pathways. Moreover, the anti-HCC effect of miR-22 was dependent on Gal-1 silencing. miR-22-high/Gal-1-low HCC patients had the best survival outcomes. In addition to the above-mentioned key intracellular pathways, miR-22 gene therapy and Gal-1 siRNA treatment of HCC reduced O-linked glycosylation, suggesting the role of the miR-22-Gal-1 axis in modifying glycosylation, which may affect the extracellular functions of Gal-1.

**Conclusion:**

In summary, the miR-22-Gal-1 axis can be an HCC prognostic biomarker, and it has vital roles in regulating metabolism and tumor immunity.

**Supplementary Information:**

The online version contains supplementary material available at 10.1186/s40364-025-00838-3.

## Introduction

Despite recent advancements in hepatocellular carcinoma (HCC) treatment, several critical knowledge gaps remain, and there is a pressing need to identify novel biomarkers for HCC that can also serve as therapeutic targets. For example, α-fetoprotein (AFP) is traditionally used to diagnose HCC, which has limitations in sensitivity and specificity [1]. Additionally, AFP is not a viable target for treatment. Targeting how HCC arises in the first place can treat HCC rather than just control cancer growth. Another significant challenge is resistance to existing therapies, and understanding the molecular mechanisms behind this resistance is crucial for developing effective treatment options.

Both miR-22 and galectin-1 (Gal-1) show significant potential as biomarkers and treatment targets for HCC. Our previous studies have demonstrated that miR-22 gene therapy and silencing Gal-1 are two potential options for HCC treatment [2–4]. Preclinical studies have shown that Gal-1 inhibition alleviates liver fibrosis and effectively prevents or treats HCC and metabolic dysfunction-associated steatohepatitis (MASH)-HCC [3]. miR-22 can be detected in body fluids, such as serum or plasma [5]. Lower serum levels of miR-22 are associated with poor prognosis, tumor progression, and angiogenesis in HCC patients. Thus, reduced miR-22 can potentially be a diagnostic and prognostic biomarker [6]. In contrast, Gal-1 is overexpressed in HCC and correlates with tumor progression, angiogenesis, and immune suppression [3, 7]. Elevated serum Gal-1 is associated with adverse outcomes in HCC patients, underscoring its potential value as a prognostic biomarker [8]. These findings suggest that miR-22 and Gal-1, together, might be a robust approach to predicting HCC, its progression, and patient outcomes.

Metabolic stimulators, such as bile acid and retinoic acid (RA), induce miR-22 through nuclear receptor-dependent mechanisms in liver and colon cancer cells [9, 10]. In contrast, inflammatory factor NF-κB and hypoxia signaling via hypoxia-inducible factor-1 (HIF-1α) stimulate Gal-1 expression [9–13]. Moreover, miR-22 silences HIF-1α, thereby miR-22 can potentially reduce Gal-1 expression via inhibiting HIF-1α [2]. Additionally, miR-22 directly silences Gal-1 by binding to its 3' UTR [14]. Consistently, Gal-1 expression levels negatively correlate with miR-22 levels in hepatic stellate cells [14]. Furthermore, the forced overexpression of Gal-1 promotes hepatic stellate cell-induced T-cell apoptosis, whereas the overexpression of miR-22 inhibits this process, revealing their opposing effects as demonstrated in co-culture experiments [14]. Taken together, miR-22 functions as a tumor suppressor in HCC, silencing Gal-1 inducer HIF-1α and Gal-1 itself. The role of miR-22 in regulating endogenous Gal-1 functions in HCC remains to be elucidated.

For the first time, the current study reveals the temporal roles of miR-22 in human HCC progression as well as the spatial effects of miR-22 in mouse HCC treatment based on histological location. We further established the significance of miR-22-Gal-1 axis by demonstrating the anti-HCC effect of miR-22 was Gal-1-dependent. Genes and pathways commonly regulated by miR-22 overexpression and Gal-1 silencing were present in both mouse and human HCC. Metabolism, complement-clotting cascades, immunity, Rho GTPase, matrix remodeling, and glycosylation, among others, are key common signaling pathways. Moreover, for the first time, we demonstrated that LLS30, a synthetic Gal-1 inhibitor, also treated mouse HCC by targeting the same signaling pathways. Using miR-22 overexpression, Gal-1 siRNA, and a small molecule to inhibit Gal-1, the presented data revealed the vital roles of the miR-22-Gal-1 axis in HCC development and treatment.

## Materials and Methods

### The Cancer Genome Atlas (TCGA) data analysis

Primary tumor specimens from the TCGA liver hepatocellular carcinoma (LIHC) dataset were analyzed using the University of California, Santa Cruz (UCSC) Xena analysis hub [3]. Samples were divided into two groups based on the upper and lower quartiles of miR-22 levels: (1) High miR-22 expression with Log2(RPM) > 17.48 (*n* = 89) and (2) Low miR-22 expression with Log2(RPM) < 16.5 (*n* = 92). Similarly, for *LGALS1*, HCCs were classified based on the upper and lower quartiles of Gal-1 mRNA levels (1) High *LGALS1* expression with Log2(RPM) > 11.92 (*n* = 102) and (2) Low *LGALS1*expression with Log2(RPM) < 10.43 (*n* = 94). Differential gene expression analysis between these groups was conducted using the UCSC Xena analysis hub [3]. In addition, the resulting data were further analyzed through Reactome-based pathway analysis to identify key biological pathways associated with variations in miR-22 and *LGALS1* mRNA levels. Significant functional pathways or processes with FDR < 0.1 and Bonferroni value < 0.1 were accepted. The DEGs and pathways related to miR-22 expression levels were also analyzed based on tumor stage, classified into three groups: (1) Stage I (*n* = 92), (2) Stage II (*n* = 60), and (3) Stages III & IV (*n* = 57). The DEGs cutoff was set at quartiles for each gene. To test if the miR-22-Gal-1 axis is associated with overall survival, we performed multivariate survival analysis of LIHC data using the DoSurvive tools [15].

### HCC models

Male and female 6-week-old FVB/N mice were from Jackson Laboratory (Sacramento, CA, USA). Orthotopic mouse HCC were produced by hydrodynamic injection of pT3-EF1α-HA-myr-AKT, pT3-EF1α-N90-pT/Caggs-Nras-v12, and pCMV-SB11 as previously described [2–4, 16]. Mice were housed, fed, and monitored according to approved protocols by the Institutional Animal Care and Use Committee (IACUC) at the University of California, Davis (Sacramento, CA, USA).

### Drug administration or treatment

An adeno-associated virus (AAV), specifically AAV8 and AAV9, was delivered by intravenous injection to express either miR-22 or *lgals1* (Gal-1) siRNA, respectively (at a dose of 5 × 10^12^ gene copies/kg, one injection). AAV8 or AAV9 blanks were used as the controls (Applied Biological Materials Inc., Richmond, BC, Canada). To overexpress Gal-1, HCC mice were treated with AAV9-Gal-1 (i.e., Gal-1 overexpression, 5 × 10^12^ gene copies/kg, one injection). LLS30, synthesized in our lab (30 mg/kg, dissolved in 8.25% alcohol and 8.25% Tween-80 in PBS) was delivered intravenously daily for three weeks [17]. Lenvatinib (10 mg/kg/day, MedChem Express, Monmouth Junction, NJ, USA) or saline was administered via oral gavage Daily for three weeks. The treatments of HCC were initiated 7 days after oncogene injection, when the liver-to-body weight ratio was doubled. OTX008 is a Gal-1 inhibitor [18], and was purchased from Cayman Chemical, Ann Arbor, Michigan, USA. OTX008 and LLS30 (S-isomer) were used to treat human HCC cells Huh7 and mouse hepatoma Hepa-1 cells (American Type Culture Collection, Manassas, Virginia, USA) and cultured based on the provided instructions.

### GeoMx® Digital Spatial Profiler (DSP) whole transcriptome and bioinformatics

Liver Sects. (4 μm) were used for whole transcriptome sequencing via the digital spatial profiler (DSP, NanoString). Morphology markers included CD45, SYTO13 nuclear stain, and Pan-cytokeratin. Slides were stained with RNAscope and GeoMx DSP RNA detection probes per the manufacturer's protocol [3]. RNA targets were associated with GeoMx barcodes, and 12 regions of interest (ROIs) per group (4 tumors, 4 Margins, and 4 non-tumor areas) were selected. The GeoMx software segmented illumination areas based on biomarkers, and UV-cleaved DSP barcodes were collected into a 96-well plate, followed by library preparation and sequencing [3, 16]. The FASTQ sequencing files were converted into digital count files using GeoMx NGS Pipeline software. The GeoMx DSP Data Analysis suite performed quality control and data analysis. Data were filtered by the limit of quantitation and normalized by the third quartile of all counts. Differentially expressed genes (DEGs) were analyzed by linear mixed effect model (LMM) analysis and Benjamini–Hochberg multiple-correction testing with a cutoff log2-transformed fold change > 0.58 and < −0.58 with -log10(*P*) > 1.3.

### RNA isolation and quantification

RNA was extracted from the livers using TRIzol Reagent (Thermo Fisher Scientific, Waltham, MA, USA), and cDNA was generated using a cDNA Kit (Applied Biosystems, Carlsbad, CA, USA). qRT-PCR was performed on a QuantStudio 5 Fast real-time PCR system using Power SYBR Green PCR master mix (Applied Biosystems). Primers were designed using Primer3 Input software version 0.4.0.

### Western blotting

Western blotting was performed based on published methods [10, 19]. Hepatic proteins (20 ~ 40 μg) were electrophoresed and subjected to immunoblotting using anti-Gal-1 antibodies (Abcam, Cambridge, MA, USA).

### Histology and Immunohistochemistry

Immunohistochemistry was performed following established protocols with specific antibodies for Gal-1 (Abcam, Cambridge, MA, USA) [3]. Cell proliferation was assessed using anti-Ki-67 antibodies (Neo Markers, Fremont, CA, USA). Counted Ki67-positive cells in at least five random microscopic fields (10X magnification) per specimen and quantified the number of proliferating cells using QuPath software [20].

### RNA sequencing and bioinformatics data analysis

RNA was quantified using a Nanodrop spectrophotometer, and RNA quality was assessed with a Qubit fluorometer and the Agilent RNA 6000 Nano Kit (Agilent Technologies, Santa Clara, CA, USA). RNA sequencing was conducted by Novogene (Sacramento, CA, USA). Read quality was assessed using FastQC (v0.11.7) and analyzed using the Salmon, tximport, and DESeq2 pipeline. Pair-ended reads (FASTQ format) were mapped to the reference genome assembly (GRCm39, GENCODE release 25) and quantified with Salmon [21]. Gene-level counts were imported with *tximport,* and differential expression analysis was performed with DESeq2 (version 1.18) with the corrected *p* value < 0.05 and fold change > 1.5 [22, 23]. Pathway analysis was conducted using the Functional Annotation Tool in the Database for Annotation, Visualization, and Integrated Discovery (DAVID) [24]. Functional pathways based on the Reactome dataset with *p* < 0.05 and Bonferroni value < 0.1 were considered significant.

### Biochemical analysis

Serum biochemical parameters, including serum aspartate aminotransferase (AST), alanine transaminase (ALT), total protein, lactate dehydrogenase (LDH), cholesterol, amylase, and creatine kinase, were measured using kits following the manufacturer's instructions.

### Statistical analysis

Statistical analysis was performed using Prism software v8.2.1 (GraphPad Software). Data were expressed as means ± standard deviation. The statistical significance between the two groups was evaluated using a two-tailed Student's t-test. A one-way ANOVA followed by Tukey's HSD test was used to compare the statistical differences among multiple groups. Associations were analyzed by linear regression. The value of *p* < 0.05 was considered statistically significant.

## Results

### The roles of miR-22 in HCC progression

**Fig. 1:**
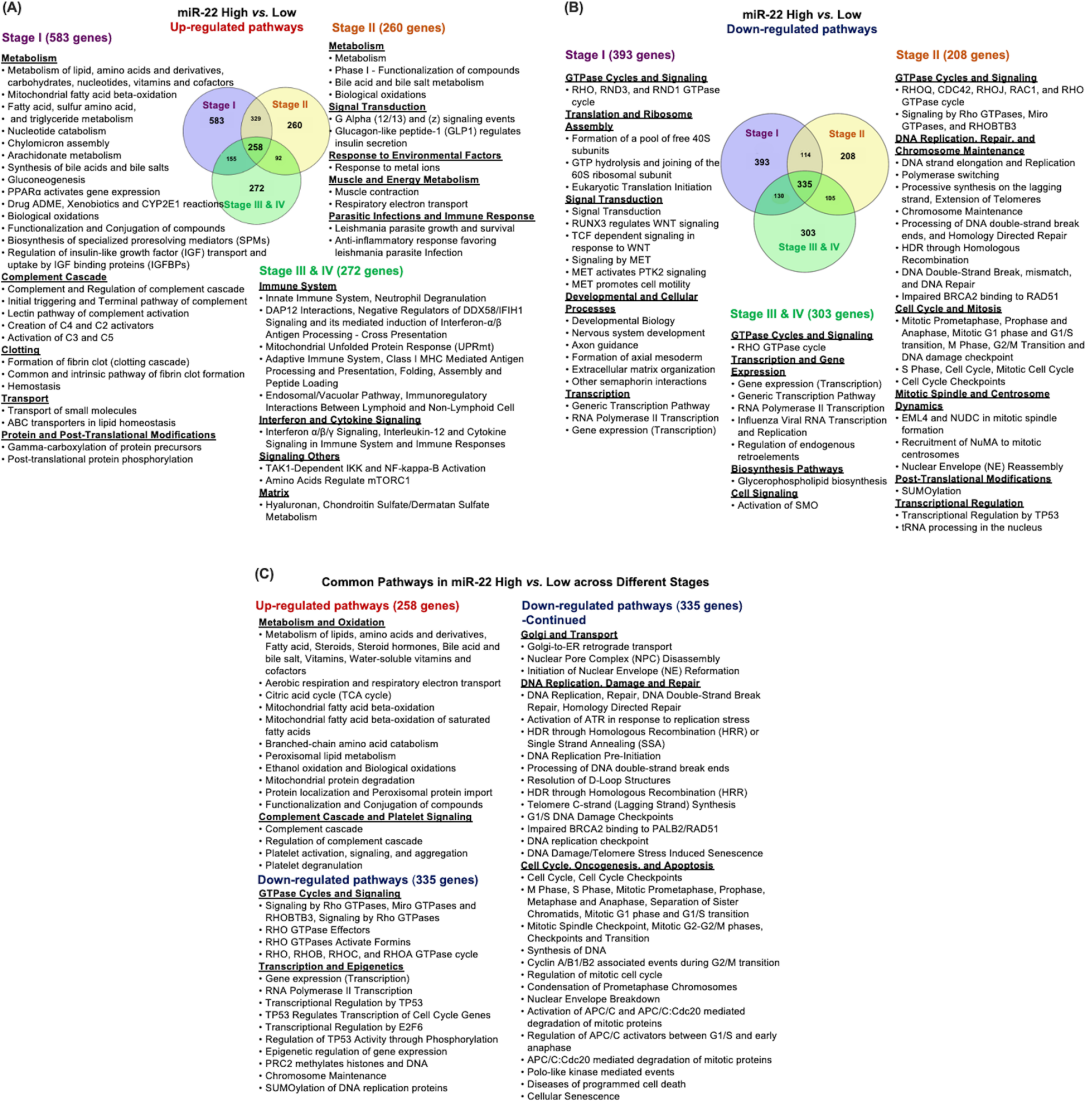
The pathways affected by miR-22 expression levels in early and late HCC stages. **A **Upregulated and (**B**) down-regulated pathways based on miR-22 expression levels in stages I, II, or III and IV HCC. **C **Common pathways in miR-22-high vs. miR-22-low in all four stages. *p* < 0.05 and a Bonferroni-adjusted *p*-value < 0.1

To investigate the temporal effects of miR-22 during HCC development, we identified molecular signatures associated with miR-22 expression levels at different stages of HCC. Stages I and II are early HCC with options for curative treatment, while stages III and IV are advanced and require systemic therapy or palliative care. Four stages of HCCs were classified into miR-22-high and miR-22-low cohorts for transcriptomics analysis. The data revealed that upregulated metabolic, complement, and coagulation pathways are key features of miR-22-high at the early stage of HCC (Fig. 1A). In advanced stages, miR-22-high HCC exhibited upregulated pathways related to immunity, interferon, and cytokine signaling (Fig. 1A). The findings suggested that increased metabolism, linked with high miR-22 expression, was accompanied by increased immunity in the later stages.

In down-regulated signaling, miR-22-high HCC had reduced GTPase cycle, WNT, and MET signaling. In the advanced stages, miR-22-high HCC exhibited reduced signaling in the GTPase cycle, DNA replication, damage repair, mitosis, and cell cycle, revealing heterogeneity of the tumors influenced by miR-22 expression level even at the same stages (Fig. 1B).

There were 258 genes commonly upregulated across all stages of HCC. Pathway analysis of those genes revealed that miR-22-high HCC consistently had increased metabolism and a complement cascade. Pathway analysis of 335 commonly downregulated genes revealed that pathways related to Rho GTPase signaling, cell cycle, and DNA replication and repair were reduced in miR-22-high HCC (Fig. 1C). These pathways are likely to be the hallmarks impacted by the differential miR-22 expression levels.

### The spatial impacts of miR-22 in mouse HCC

**Fig. 2:**
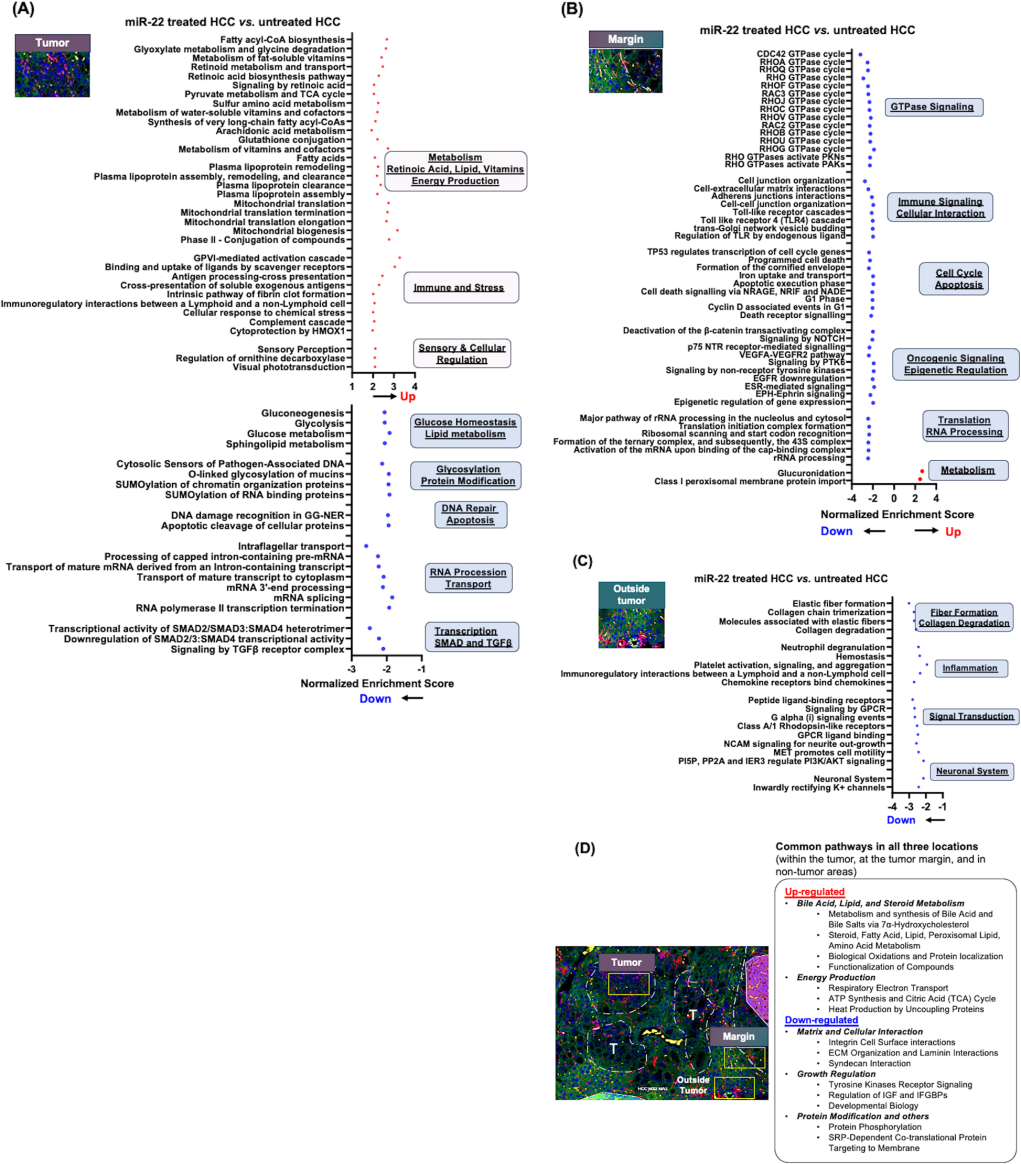
The spatial effects of miR-22 in mouse HCC treatment. Pathways affected by miR-22 treatment in HCC were identified in different locations by analyzing spatial transcriptomics data. **A** Within the tumor, **B **at the tumor margin, **C **in non-tumor areas, and (**D**) pathways commonly found in three locations (within the tumor, at the tumor margin, and in non-tumor regions)

**Fig. 3:**
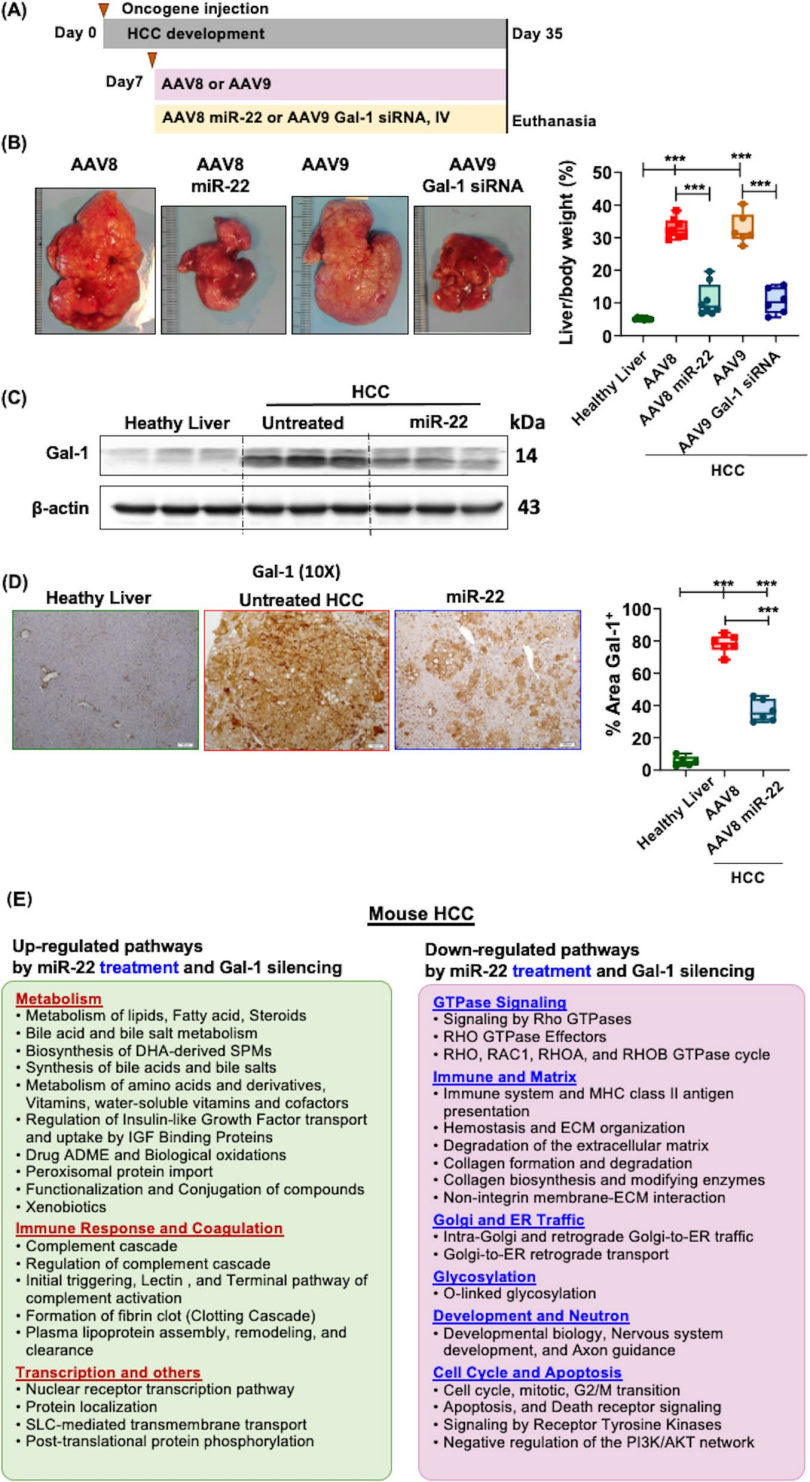
Pathways regulated by miR-22 treatment and Gal-1 silence in mouse HCC. **A **Study design for miR-22 or Gal-1 siRNA treatment in RAS/AKT-induced HCC model. One week after the tumor initiation, miR-22 or *Lgals1* siRNA (5 × 10^12^ gene copies/kg) was administered intravenously. **B **Representative liver morphology and liver-to-body weight ratio of miR-22 or *Lgals1* siRNA-treated liver. **C**-**D **Gal-1 protein levels were assessed using Western blot (**C**) and immunohistochemistry image (**D**), and % area of Gal-1^+^ measurement (**D**) in healthy liver tissues, untreated HCC, and miR-22-treated HCC. *n* = 6–8 mice/group. ∗ *p* < 0.05, ∗ ∗ *p* < 0.01, ∗ ∗ ∗ *p* < 0.001 by one-way ANOVA with Tukey's multiple comparison tests. (E) Pathways involving genes regulated by miR-22 and *Lgals-1* siRNA were analyzed using bulk transcriptomics analysis of mouse HCC treated with miR-22 and *Lgals-1* siRNA. *p* < 0.05 and a Bonferroni-adjusted *p*-value < 0.1

We further analyzed the spatial effects of miR-22 in HCC. Mouse HCC was treated with miR-22-AAV8, followed by spatial transcriptomics analysis to map the location-specific effects of miR-22 in liver sections (Fig. 2). miR-22 treatment had an anti-HCC impact, and the outcome was consistent with our previous report, summarized below (Fig. 3) [2].

The spatial transcriptomic data mapped miR-22-induced metabolism inside the tumors. The improved metabolism encompassed fatty acids, amino acids, fat-soluble vitamins, pyruvate, the tricarboxylic acid (TCA) cycle, and mitochondrial biogenesis (Fig. 2A). Interestingly, increased retinoid metabolism and transport, as well as RA biosynthesis, were among the most significant signaling pathways within the tumors (Fig. 2A). RA is a known inducer of miR-22, which aligned with our previous finding that RA is involved in both upstream and downstream regulation of miR-22 signaling [9]. Additionally, miR-22 treatment induced the complement cascade, intrinsic pathways for fibrin clot formation, and antigen presentation inside the tumors (Fig. 2A). miR-22 treatment also inhibited specific signaling within the tumor. Among them, transcription regulation mediated by SMAD and TGFβ, RNA processing, glycolysis, and gluconeogenesis were among the most significantly downregulated pathways (Fig. 2A).

At the tumor margin, miR-22 inhibited Rho GTPase signaling, toll-like receptor signaling, trans-Golgi network budding, cell interactions, VEGF, EGFR signaling, and cell cycle signaling, among others. However, glucuronidation and the class I peroxisomal membrane protein import pathways were the only two pathways upregulated in response to miR-22 treatment at the tumor margin, revealing location-specific effects (Fig. 2B).

In the non-tumor areas, miR-22 inhibited collagen degradation, neutrophil degranulation, platelet activation, chemokine signaling, and G-protein coupled receptor ligand binding (Fig. 2C). These findings suggested the anti-fibrotic and anti-inflammatory effects of miR-22 in non-tumor areas. Common pathways in response to miR-22 treatment across all three locations were upregulated lipid and amino acid metabolism, as well as ATP energy production. In response to miR-22 gene therapy, three Locations consistently exhibited increased bile acid synthesis, mediated by 7α-hydroxylation, and induced steroid metabolism (Fig. 2D). It has been shown that dysregulated bile acid synthesis contributes to liver carcinogenesis [25–28], and miR-22 was able to reverse the carcinogenesis processes.

The commonly downregulated pathways in all three locations included matrix remodeling, integrin cell surface interaction, and laminin interaction, as well as growth signaling pathways such as IGF and tyrosine kinase receptor (Fig. 2D). Thus, miR-22 not only improves parenchymal cell metabolism but also has an impact on the functions of mesenchymal cells, covering stellate cells and portal fibroblasts.

### Treatment with miR-22 or Gal-1 silencing reduced tumor burden in HCC

miR-22 silences Gal-1, which is abundantly expressed in human and mouse HCC [3, 14, 29]. We compared molecular signatures in response to miR-22 or Gal-1 siRNA treatments. The treatments were initiated after the tumors had developed at 7 days post-oncogene injection (Fig. 3A). Both miR-22 treatment and Gal-1 siRNA significantly reduced the tumor Load, as measured by the liver-to-body weight ratio, to 9% compared with 30% in the untreated control group (Fig. 3B). Consistent with reduced tumor load, Gal-1 expression, low in healthy livers, was markedly elevated in HCC but significantly reduced in response to miR-22 treatment, as shown by Western blot and immunohistochemical (IHC) (Fig. 3C-D).

### Pathways affected by miR-22 expression or Gal-1 silencing in HCC

Transcriptomic profiling revealed the shared roles of miR-22 treatment and Gal-1siRNA (Fig. 3E). Both treatments improved metabolism, encompassing the upregulation of bile acid, fatty acid metabolism, and vitamin metabolism, as well as the complement and clotting cascades. The Rho GTPase cycle, immune and vascular responses, matrix remodeling, O-linked glycosylation, cell cycle, and Golgi and ER traffic were the most significantly downregulated signaling pathways commonly regulated by miR-22 overexpression and Gal-1 siRNA (Fig. 3E).

Common genes, regulated by miR-22 treatment and Gal-1 siRNA, involved in complement cascades and clotting, Rho GTPase, O-linked glycosylation pathways, and their cancer relevance are summarized in Table 1. Specifically, miR-22 treatment increased the expression of *C1r* (a regulator of the classical pathway) [30], *C8b*, and *C8g* (components of the membrane attack complex) [31, 32], *Masp1* and *Masp2* (serine proteases activating the lectin pathway) [35], as well as *Mbl2* (mannose-binding lectin) suggests an ongoing activation of multiple complement pathways [36]. Moreover, the induction of *Cfh* (complement factor H) and *Cfhr1* (complement factor H-related protein 1) indicates that regulatory mechanisms are also engaged [37, 38], potentially limiting complement-mediated damage. It is important to emphasize that Gal-1 is an immune suppressor [3, 7, 59]. miR-22-silenced Gal-1, likely via activation of the complement and coagulation systems, to initiate an inflammatory response to facilitate tumor destruction. This scenario is supported by data showing an increased class I MHC-mediated antigen presentation, adaptive immunity, and interferon signaling noted in miR-22-high advanced HCC.

**Table 1:** The differentially expressed genes in the (A) Complement cascade, (B) GTPase cycle signaling, and (C) O-linked glycosylation pathways in healthy livers, mouse HCC, and miR-22- or Gal-1 siRNA-treated HCC. Their relevance to human HCC is summarized

(A) *Complement cascade and clotting*						
Genes	Mouse HCC			Human HCC		
	HCC *vs* Healthy	miR-22 *vs* Untreated HCC	Gal-1 siRNA *vs* Untreated HCC	Tumor vs non-tumor	References	Prognostic (https://www.proteinatlas.org/)
* C1r*	**↓**	**↑**	**↑**	**↓**	[30]	High, Favorable
* C8b*	**↓**	**↑**	**↑**	**↓**	[31]	High, Favorable
* C8g*	**↓**	**↑**	**↑**	**↓**	[32]	High, Favorable
* Hc (C5)*	**↓**	**↑**	**↑**	**↓**	[33]	Unknown
* Colec10*	**-**	**↑**	**↑**	**↓**	[34]	High, Favorable
* Masp1*	**↓**	**↑**	**↑**	**↓**	[35]	High, Favorable
* Masp2*	**↓**	**↑**	**↑**	**↓**		High, Favorable
* Mbl2*	**↓**	**↑**	**↑**	**↓**	[36]	High, Favorable
* Cfh*	**↓**	**↑**	**↑**	**↓**	[37]	Unknown
* Cfhr1*	**↓**	**↑**	**↑**	**↓**	[38]	High, Favorable
* Cpn2*	**↓**	**↑**	**↑**	**↓**		High, Favorable
(B) *Rho GTPase signaling*						
Genes	Mouse HCC			Human HCC		
	HCC *vs* Healthy	miR-22 *vs* Untreated HCC	Gal-1 siRNA *vs* Untreated HCC	Tumor *vs*. non-tumor	References	Prognostic (https://www.proteinatlas.org/)
* Arhgef2*	↑	↓	↓	↑	[39]	High, Unfavorable
* Arhgef16*	↑	↓	↓	-		High, Unfavorable
* Vav3*	↑	↓	↓	Unknown	Unknown	Unknown
* Net1*	↑	↓	↓	↑	[40, 41],	High, Unfavorable
* Ect2*	↑	↓	↓	↑	[42]	High, Unfavorable
* Arhgap44*	↑	↓	↓	Unknown		Unknown
* Arhgap22*	↑	↓	↓	↑		Unknown
* Racgap1*	↑	↓	↓	↑	[43]	High, Unfavorable
* Rhoj*	↑	↓	↓	Unknown		Unknown
* Cdc42ep1*	↑	↓	↓	↑	[44]	Unknown
* Pak3*	↑	↓	↓	↑	[45]	Unknown
* Pak6*	↑	↓	↓	↑	[46]	Unknown
* Iqgap3*	↑	↓	↓	↑	[47]	High, Unfavorable
* Diaph3*	↑	↓	↓	↑	[48]	High, Unfavorable
* Cyfip2*	↑	↓	↓	Unknown		Unknown
* Prag1*	↑	↓	↓	Unknown		Unknown
* Rtkn*	↑	↓	↓	↑	[49]	Unknown
(C) *O-linked glycosylation*						
Genes	Mouse HCC			Human HCC		
	HCC *vs* Healthy	miR-22 *vs* Untreated HCC	Gal-1 siRNA *vs* Untreated HCC	Tumor *vs*. non-tumor		Prognostic (https://www.proteinatlas.org/)
* B4galt6*	↑	↓	↓	↑	[50]	Unknown
* Large2*	↑	↓	↓	Unknown		Unknown
* St3gal2*	↑	↓	↓	↑	[51]	High, Unfavorable
* Galnt10*	↑	↓	↓	↑	[52]	Unknown
* Spon1*	↑	↓	↓	↑	[53]	Unknown
* Spon2*	↑	↓	↓	↑	[54, 55]	Unknown
* Adamts1*	↑	↓	↓	↑	[56]	Unknown
* Adamts4*	↑	↓	↓	Unknown		Unknown
* Adamts12*	↑	↓	↓	↑	[57]	Unknown
* Adamts15*	↑	↓	↓	↑	[58]	Unknown

In the Rho GTPase signaling, the GTPase genes inhibited by miR-22 treatment and Gal-1 siRNA were *Arhgef2, Arhgef16, Vav3*, *Net1,* and *Ect2,* which are guanine nucleotide exchange factors (GEFs) (Table 1B) [37, 39–42]. Genes *Arhgap22*, *Arhgap44, Racgap1*, *Rhoj*, *Cdc42ep1* are GTPase-activating proteins (GAPs), and *Pak3*, *Pak6*, *Iqgap3, Diaph3*, *Cyfip2*, *Prag1,* and *Rtkn* are the effectors [43–46, 48]. Additionally, GEFs (*Ect2*, *Vav3*, and *Arhgef2*), GAPs (*Racgap1* and *RhoJ*) [42], and effectors (*Pak3*, *Rtkn*, and *Iqgap3*) are often overexpressed in HCC [45, 47, 49, 60]. The simultaneous inhibition of GEFs, GAPs, and effectors suggests a broad inhibition of Rho GTPase signaling, which is critical for tumor progression [61].

Genes affected by miR-22 treatment and Gal-1 siRNA in the O-linked glycosylation included *B4galt6*, *Large2, St3gal2*, *Galnt10, Spon1*, *Spon2*, *Adamts1, Adamts4, Adamts12, Adamts15* (Table 1C). These intracellular gene expression changes likely extend to extracellular Gal-1 and glycan interactions.

### Pathways affected by miR-22 and Gal-1 expression levels in human HCC

**Fig. 4:**
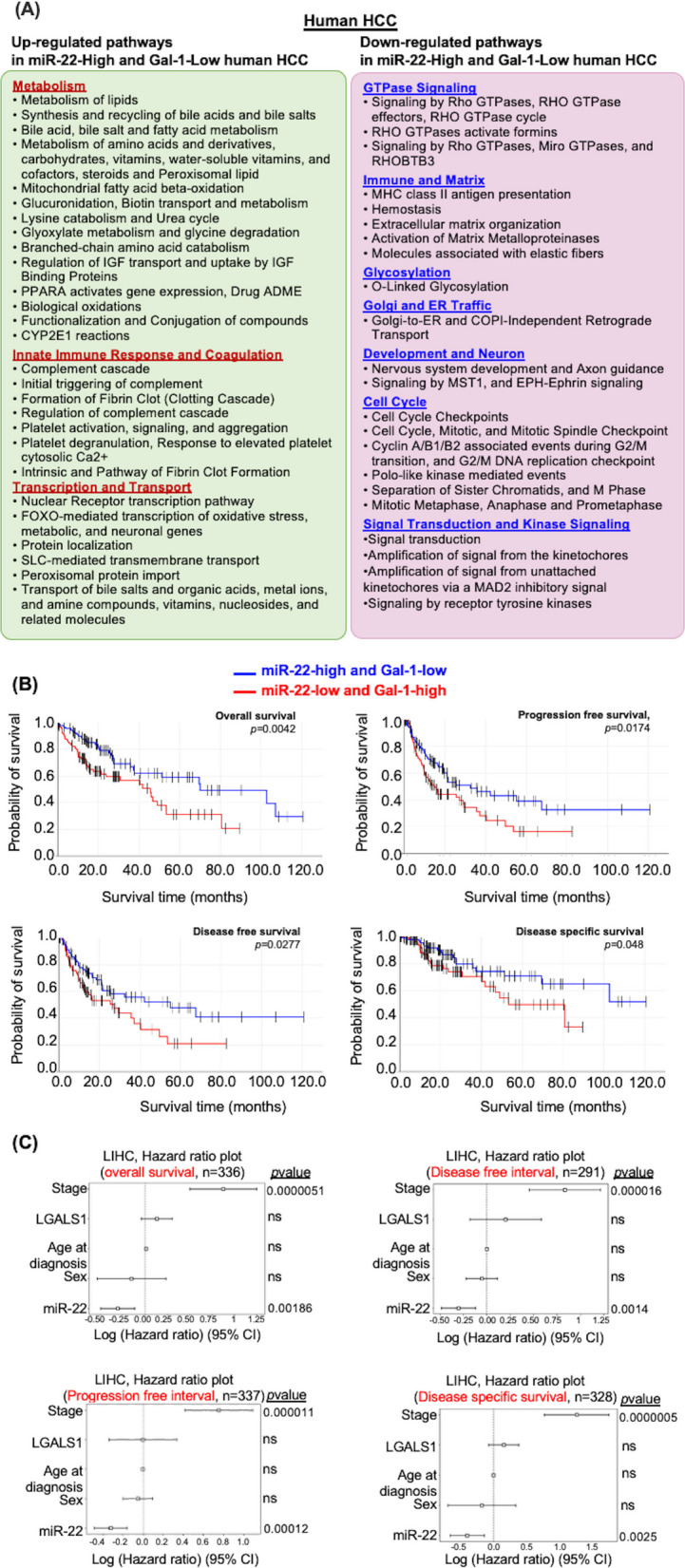
The levels of miR-22 and Gal-1 mRNA affect the expression levels of genes and pathways in human HCC. **A **Common pathways were identified by comparing miR-22-Hi (high, *n* = 89) with miR-22-Lo (low, *n* = 92) and *LGALS1*-Lo (Low, *n* = 94) with *LGALS1*-Hi (High, *n* = 102) in human HCC. *p* < 0.05 and Bonferroni-adjusted *p*-value < 0.1. **B **miR-22 and *the LGALS1* gene are prognostically significant in HCC. Kaplan–Meier survival analysis of combined expressions of miR-22 and *LGALS1* in HCC patients. **C **Cox regression analysis of miR-22 and *LGALS1* using TCGA LIHC data to study overall survival (*n* = 336), disease-free interval (*n* = 291), progression-free interval (*n* = 337), and disease-specific survival (*n* = 328)

To assess the human relevance of findings from mouse models, we identified pathways that were differentially expressed based on miR-22 and Gal-1 levels in human HCC (Fig. 4A). Using the TCGA LIHC database, HCCs were grouped into 89 miR-22-high and 92 miR-22-low cases, as well as 102 Gal-1-high and 92 Gal-1-low HCCs. Common pathways identified in miR-22-high and Gal-1-low HCC included endobiotic metabolism and xenobiotic detoxification, the complement cascade, and clotting signaling, among others (Fig. 4A). The down-regulated pathways in miR-22-high and Gal-1-low HCC included the GTPase cycle, matrix remodeling, O-linked glycosylation, Golgi and ER trafficking, and the cell cycle (Fig. 4). Thus, the data aligned with findings from mouse models.

We further analyzed the combined effects of miR-22 and Gal-1 by comparing whether the miR-22-high/Gal-1 low state remains associated with favorable survival outcomes using bivariate Kaplan–Meier survival analysis (Fig. 4B). Group comparison between miR-22-high/Gal-1-low and miR-22-low/Gal-1-high exhibited significant differences in all four survival outcome analyses, including overall, disease-free, progressive-free, and disease-specific survival (Fig. 4B). Comparison between miR-22-high/Gal-1-low and miR-22-low/Gal-1-low cohorts showed that the former group had better progressive and disease-free outcomes suggesting the benefits of having high miR-22. Other comparisons did not show significant differences (Table 2). The data support that the miR-22-Gal-1 axis can be a potential prognostic biomarker.

**Table 2:** The survival outcomes of miR-22-high/Gal-1-low HCC *vs*. other cohorts with differential miR-22 and Gal-1 expression levels using TCGA-LIHC data

Comparison: miR-22-high/Gal-1-low *vs.* miR-22-low/Gal-1-high		
Survival Type	Number of Patients	*p*value
Overall	191	0.0042
Progression-Free	191	0.0174
Disease-Free	162	0.0277
Disease-Specific	186	0.0488
Comparison: miR-22-high/Gal-1-low ***vs***. miR-22-low/Gal-1-low		
Survival Type	Number of Patients	*p*value
Overall	181	Not Significant
Progression-Free	181	0.0313
Disease-Free	156	0.0103
Disease-Specific	178	Not Significant
Comparison: miR-22-high/Gal-1-low ***vs***. miR-22-high/Gal-1-high		
Survival Type	Number of Patients	*p*value
Overall	184	Not Significant
Progression-Free	184	Not Significant
Disease-Free	158	Not Significant
Disease-Specific	180	Not Significant

By including stage, age at diagnosis, sex, and the expression levels of miR-22 and Gal-1, Cox regression analysis plots showed that miR-22 was a protective factor. In contrast, stage was a risk in overall, disease-free, and progression-free survival (Fig. 4C, Supplementary Table 1). The data suggested that miR-22, which inhibits many cancer pathways, including Gal-1, had a more significant impact on HCC survival outcomes.

### The anti-HCC effect of miR-22 is Gal-1-dependent

**Fig. 5:**
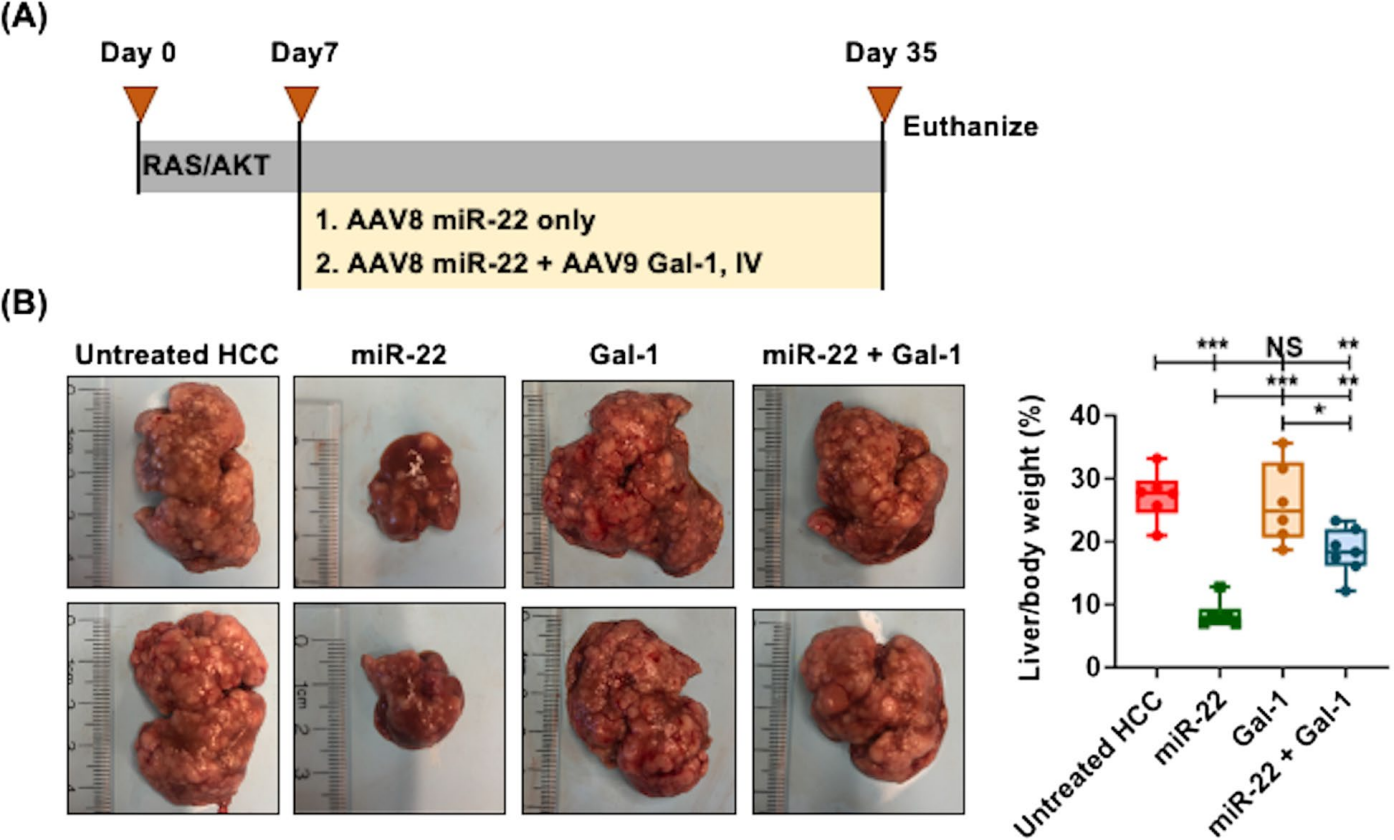
The anti-HCC effect of miR-22 is Gal-1-dependent. **A **Study design for RAS/AKT-induced mouse HCC treated with miR-22 with or without Gal-1 overexpression. One week after RAS and AKT injection, miR-22 or AAV9 Gal-1 (5 × 10^12^ gene copies/kg) was administered intravenously. **B **Representative liver morphology and (**C**) the liver weight-to-body weight ratios of the mouse model of HCC, untreated, treated with miR-22, treated with Gal-1 overexpression, and treated with a combination of miR-22 and Gal-1 overexpression (*n* = 6/group). ∗ *p* < 0.05, ∗ ∗ *p* < 0.01, ∗ ∗ ∗ *p* < 0.001 by one-way ANOVA with Tukey's multiple comparison tests

To study the significance of Gal-1 silencing in contributing to the anti-HCC effects of miR-22, we investigated whether delivery of AAV9-Gal-1, which was already highly expressed in HCC, would attenuate the anti-HCC effects of miR-22. The experimental design schema is shown in Fig. 5A. The data showed that forced expression of additional Gal-1 indeed reduced the HCC treatment effects of miR-22. While miR-22 treatment reduced the liver-to-body weight ratio to 8%, the ratio was 18% in the presence of AAV9-Gal-1, partially inhibiting the anti-HCC effect of miR-22 (Fig. 5B).

### Pharmacological inhibition of Gal-1 treats HCC

**Fig. 6:**
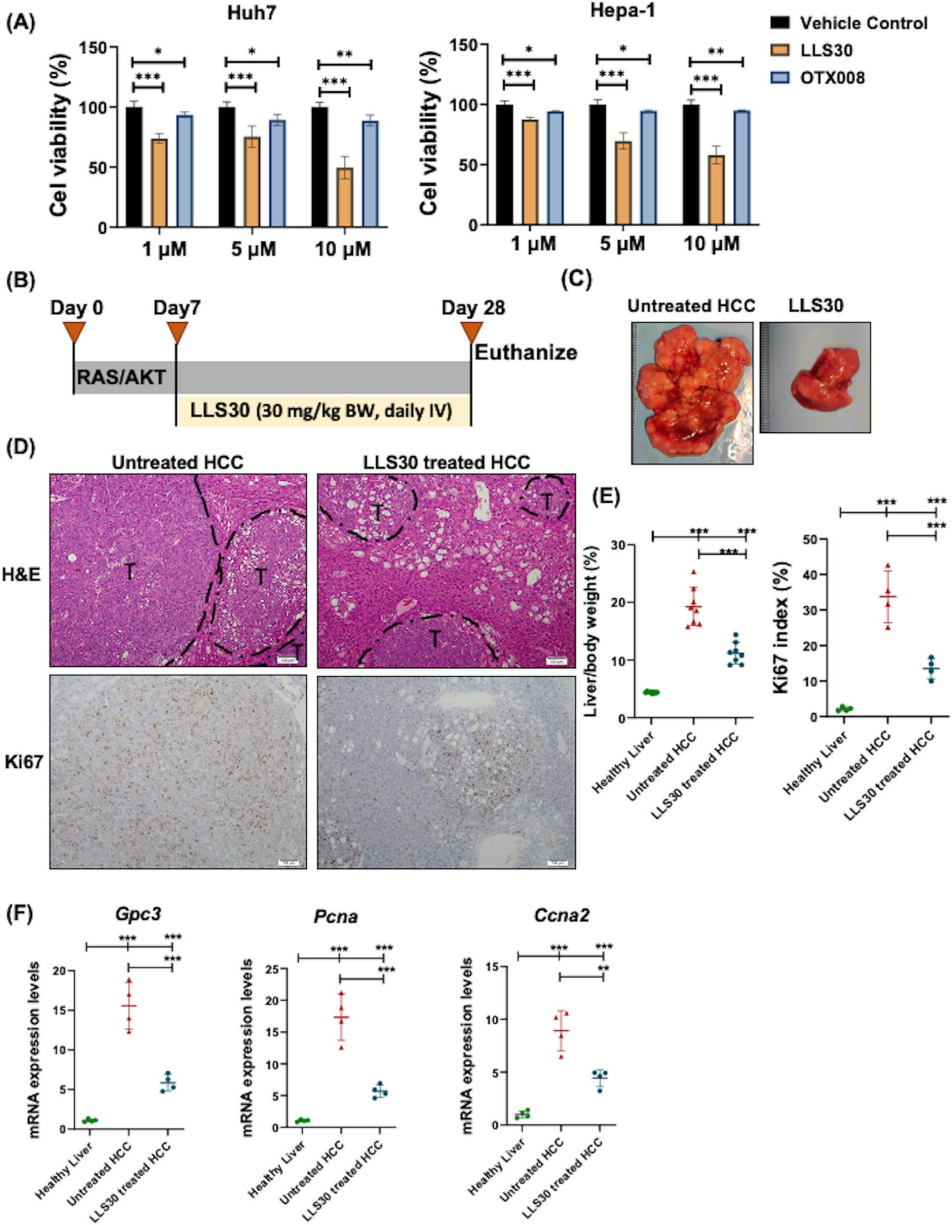
Gal-1 inhibitor LLS30 treats mouse HCC. **A **Huh7 and Hepa-1 cell viability in response to LLS30 or OTX008 treatment at different doses after 48 h of treatment. **B **Study design of RAS/AKT-induced mouse HCC treated with LLS30. **C **Representative liver morphology, **D **H&E-stained liver sections, and Ki67 IHC staining of untreated HCC and HCC mice treated with LLS30. **E **The liver-to-body weight ratios, Ki67 index (*n* = 4–8/group), and (**F**) Hepatic mRNA levels of *Gpc3*, *Pcna, and Ccna2* of healthy mice, untreated HCC mice, and HCC mice treated with LLS30 (*n* = 4–8/group). Data represent mean ± SD. ∗ *p* < 0.05, ∗ ∗ *p* < 0.01, ∗ ∗ ∗ *p* < 0.001 by one-way ANOVA with Tukey's multiple comparison tests

Given the critical role of Gal-1 in HCC, there is an urgent need to develop effective Gal-1 inhibitors. We initially evaluated the anti-HCC effects of LLS30 and OTX008 using Huh7 and Hepa-1 liver cancer cells (Fig. 6A). Both are small molecules that are expected to penetrate cells to assess the intracellular effects of Gal-1 [17, 62]. LLS30 exhibited a superior anti-proliferative effect compared to OTX008 (Fig. 6A). A Phase I trial of OTX008 reported dose-limiting toxicities that required halting dose escalation, including skin ulcerations and neurological adverse effects [63]. Thus, we used LLS30 to study its anti-HCC effect in preclinical models. LLS30 is an experimental drug with Ga-1 inhibitory properties, but we cannot rule out its other effects. The experimental schema for animal treatment is shown in Fig. 6B. LLS30 (30 mg/kg, administered intravenously daily for three weeks) significantly reduced tumor burden and decreased the number of Ki-67-positive cells (Fig. 6C-E). In addition, LLS30 reduced the expression of the HCC marker gene *Gpc3* and the proliferation-related genes *Pcna* and *Ccna2* at the mRNA level (Fig. 6F)**.**

**Fig. 7:**
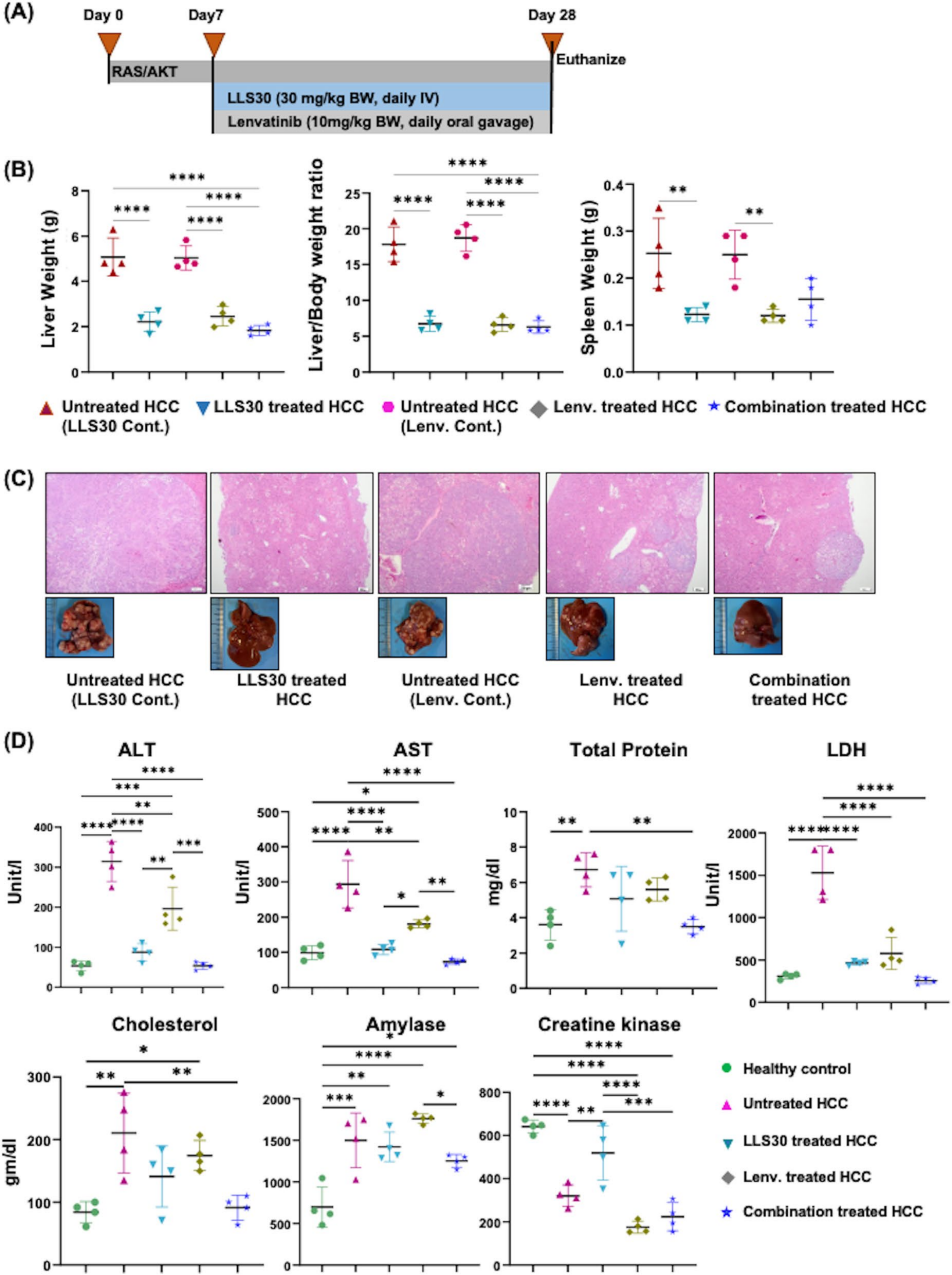
LLS30 and Lenvatinib have comparable anti-HCC effects. **A **Experimental schema; **B **Liver weight, the liver-to-body weight ratio and spleen weight (*n* = 4/group), (B) H&E staining (4X magnification) and the gross liver pictures of untreated HCC mice, HCC mice treated with either LLS30 (30 mg/kg body weight, daily I.V.) or Lenvatinib (10 mg/kg body weight, daily oral gavage), or a combination of LLS30 and Lenvatinib; **C **Measurements of biochemical markers in the serum of healthy control, untreated HCC, HCC mice treated with LLS30, Lenvatinib, and a combination of LLS30 and Lenvatinib (*n* = 4/group). Data represents ± SD. ∗ *p* < 0.05, ∗ ∗ *p* < 0.01, ∗ ∗ ∗ *p* < 0.001 by one-way ANOVA with Tukey's multiple comparison tests

We have compared the anti-HCC effect of LLS30 with Lenvatinib, the standard treatment for HCC. LLS30 and Lenvatinib had comparable treatment outcomes, based on tumor load, spleen weight, and histology (Fig. 7A-C). However, LLS30 was more effective in lowering serum ALT and AST levels than Lenvatinib (Fig. 7D). Moreover, Lenvatinib markedly reduced creatine kinase levels, suggesting potential toxicity (Fig. 7D). A combination of LLS30 and Lenvatinib did not generate improved outcomes compared to single treatments (Fig. 7).

**Fig. 8:**
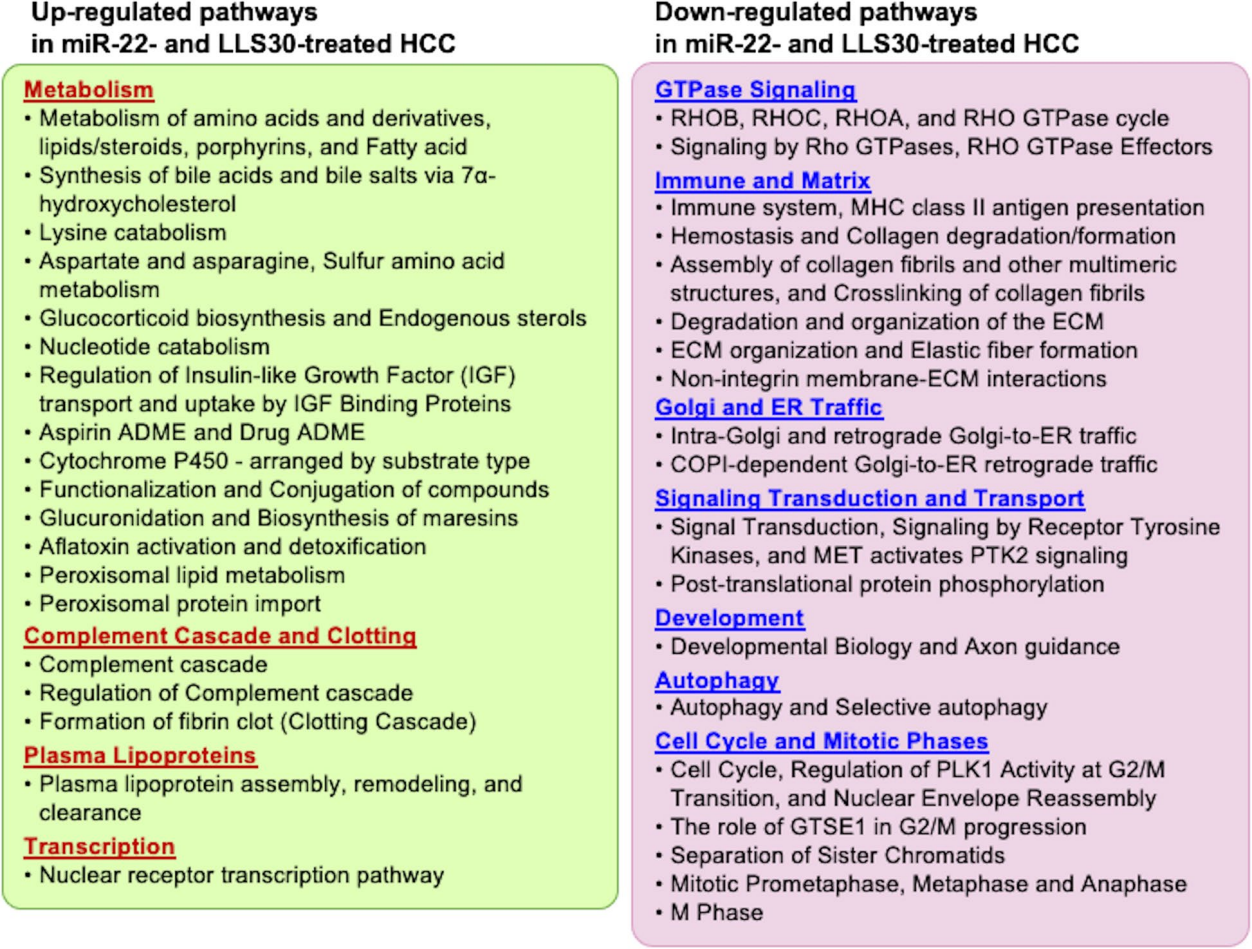
Up and down-regulated pathways commonly regulated in response to miR-22 gene therapy and LLS30 treatment in mouse HCC. Pathways commonly regulated by both miR-22 and LLS30 treatment, identified by RNA sequencing (n = 4/group). *p* < 0.05 and Bonferroni-adjusted *p*-value < 0.1

We further analyzed and compared the pathways affected by LLS30 and miR-22 treatments (Fig. 8). Both treatments induced endobiotic metabolism and xenobiotic detoxification, regulated by nuclear receptors or peroxisomal functions, as well as complement and clotting cascades. In addition, both treatments inhibited the Rho GTPase cycle, as well as matrix and collagen remodeling, cell cycle progression, and other signaling pathways (Fig. 8).

## Discussion

The presented data revealed the roles of miR-22 in human HCC progression and its spatial impacts on HCC treatment in mouse models. The data established the extensive metabolic benefits of miR-22 in the early stage of HCC within the tumors, accompanied by increased complement and clotting cascades. At the tumor margin, miR-22 inhibited Rho GTPase and cell–matrix interaction. Outside the tumors, miR-22 treatment profoundly reduces inflammatory responses by inhibiting neutrophil degranulation, platelet activation, and chemokine receptor binding, as well as fibrosis.

miR-22 has many downstream targets, and it would be relatively challenging to evaluate their relative significance in HCC treatment quantitatively. The importance of Gal-1 in carcinogenesis has been established in many types of cancer. Moreover, forced expression of Gal-1 reduced the anti-HCC effects of miR-22. Furthermore, miR-22-high/Gal-1-low HCC had favorable survival outcomes, stressing the significance of the miR-22-Gal-1 axis in HCC prognosis. Carcinogenesis consists of multi-step processes involving numerous pathways, and effective cancer treatment requires multiple drugs to target these pathways, which can lead to toxic side effects. Having a single drug, like miR-22, delivered using a single injection to target multiple cancer-specific pathways selectively, would represent an attractive approach in cancer treatment.

The study further analyzed the significant miR-22-Gal-1 axis in HCC treatment by comparing the effects of increased miR-22 and Gal-1 silencing, as well as Gal-1 inhibition using LLS30, a small molecule. Each of those approaches generated anti-HCC effects. Moreover, the anti-HCC effect of miR-22 is dependent on Gal-1. Furthermore, all three approaches target similar signaling pathways. The most significant shared pathways include activation of metabolism and detoxification, as well as complement and clotting cascades. Additionally, Rho GTPase activity, matrix remodeling, O-linked glycosylation, the cell cycle, and Golgi-ER traffic are among the most significant pathways downregulated in response to miR-22 treatment and Gal-1 inhibition.

The improved metabolism and upregulation of complement and clotting cascades found in miR-22-high HCC are noted in early stages I and II. In complement cascades, miR-22 treatment and Gal-1 silencing upregulated the expression of genes *C1r, C8b, C8g, Hc (C5), Colec10, Masp1, Masp2, Mbl2, Cfh, Cfhr1,* and *Cpn* (Table 1A). The complement system plays a dual role in cancer biology. On the one hand, it can facilitate immune-mediated destruction of tumor cells, contributing to anti-tumor immunity [64]. On the other hand, tumor cells can hijack complement activation to promote tumor growth, angiogenesis, and immune evasion [65]. Because the upregulation of the complement cascade is linked with positive treatment outcomes, it implies the benefits of miR-22 in anti-tumor immunity.

Using Gal-1 knockout and knock-in models, our data demonstrated the critical role of Gal-1 in regulating Rho GTPase, extracellular matrix, and cellular senescence in MASH-HCC mice [3]. The Rho family of GTPases belongs to the Ras superfamily of proteins, which comprises over 150 varieties in mammals. Rho GTPases are pivotal molecular switches that orchestrate actin cytoskeleton remodeling, influencing cell shape, adhesion, and motility [66–68]. They also interact with signaling pathways involved in immune responses, affecting the migration and activation of immune cells [69, 70]. Since downregulation is primarily observed at the tumor margin, inhibiting GTPase may contribute to restricting tumor expansion and immune cell migration. Together, those molecular hallmarks play key roles in shaping the tumor immune microenvironment by influencing the function and trafficking of immune cells through cytoskeletal regulation [39, 40]. Functional analysis at tumor margins using pharmacologic perturbation or ROCK inhibitors could further test the convergence on this axis, which will be the next step.

Inhibiting the O-linked glycosylation pathway in response to miR-22 treatment occurred within the tumor based on the transcriptomic data. Among those down-regulated genes (Table 1C), *B4galt6* (β−1,4-galactosyltransferase 6) catalyzes the transfer of galactose to N-acetylglucosamine, contributing to glycan synthesis [50]. *Large2* is is large xylosyl-and glucuronyltransferase, a bifunctional glycosyltransferase involved in the O-linked glycosylation of proteins [71]. The *St3gal2,* encoding a sialyltransferase that adds sialic acid to glycan chains, and *Galnt10* is a N-acetylgalactosaminyl transferase that initiates O-glycosylation [72, 73]. *Spon1 and Spon2* are ECM-associated proteins involved in cell adhesion and matrix interactions, whereas *Adamts1*, *4, 12,* and *15* are metalloproteinases involved in matrix remodeling, processing proteoglycans, and influencing tissue structure [53, 55–58, 74]. Thus, *B4galt6, Large2, St3gal2*, and *Galnt10* have pivotal roles in modifying glycoproteins. Because Gal-1 can function extracellularly, these changes in O-glycosylation might affect the extracellular functions of Gal-1. Alternatively, cytosolic galectins are known to interact with glycans that are initially confined in the luminal space of intracellular organelles, such as lysosomes, but become exposed to the cytosol when these organelles are damaged [75]. The function of Gal-1 through this pathway may be affected due to O-glycosylation changes.

It is essential to emphasize that the presented data pertain to miR-22 and Gal-1-regulated intracellular signaling pathways, as determined through transcriptomic analyses in both mouse and human models. As a β-galactoside-binding protein, Gal-1 modulates cell adhesion, migration, immune regulation, and tissue remodeling extracellularly [76, 77]. Additionally, Gal-1 influences vascular responses by promoting angiogenesis and vascular stability, which are essential during tissue repair and tumor progression [78]. Gal-1 also participates in ECM remodeling by interacting with matrix components and influencing cell motility and invasion [79]. Since some of these cellular process changes were also noted using transcriptomic data generated using gene therapy, this suggests the potential of coordinated regulation of intracellular and extracellular processes dictated by the miR-22-Gal-1 axis.

LLS30, by inhibiting Gal-1, treats castration-resistant prostate cancer progression and invasion in animal models [17]. Gal-1 inhibitors are a promising treatment option for cancer. However, the phase 1 clinical trials of OTX008, completed in 2013, did not progress to subsequent phases [63]. Many challenges remained for targeting Gal-1 or other galectins, including toxicity or specificity [80]. Perhaps miR-22 or LLS30 could be considered alternatives for the HCC [4].

The miR-22 exerts multifaceted effects. In addition to Gal-1, miR-22 has many other targets [2, 9, 10, 81, 82]. miR-22 silences cyclin A2 and multiple histone deacetylases (HDACs), such as HDAC1 and HDAC4, all highly expressed in HCC [10]. Thus, miR-22 might regulate metabolism by reducing chromatin compaction, thereby enhancing the expression of genes involved in metabolism and detoxification. It is interesting to note that RA, bile acids, and HDAC inhibitors are inducers of miR-22. Conversely, increased miR-22 induces the RA and bile acids signaling and silences HDAC, thereby forming positive feedback loops [9]. Furthermore, RXRα is an essential partner of FXR, the bile acid receptor, in regulating metabolism and immunity [83–85]. These findings highlight the importance of maintaining metabolism, which in turn promotes anti-tumor immunity. miR-22-Gal-1 is an integral signaling molecule regulating both metabolism and immunity.

AAV8/9 offer strong liver tropism and have shown robust hepatic transduction in preclinical and some clinical contexts [86]. Although both serotypes efficiently transduce hepatocytes after systemic delivery in many species, their relative efficiency among non-parenchymal liver cells might differ depending on the species, age, disease state, and delivery route [87]. Thus, one potential limitation of the current study was using two serotypes to deliver miR-22 and Gal-1 siRNA. For HCC gene therapy, achieving selective, durable, and safe delivery to tumor cells within a diseased liver is a major clinical and translational challenge. Potential hurdles include immunogenicity. For example, patients may have preexisting neutralizing antibodies against common AAV serotypes, which can block vector entry into liver cells. In addition, capsid proteins may induce cellular and humoral responses that clear transduced cells or cause inflammation. Furthermore, repeat administration of the same AAV serotype is often ineffective or unsafe. Alternatives could be using different serotypes, immunomodulation, or non-viral approaches, such as LLS30. Nevertheless, the animal trials we conducted used a single injection of AAV8 or AAV9. The positive outcomes have been demonstrated in several animal models, including RAS/AKT mutated HCC, β-catenin-positive HCC, and MASH-HCC, and we did not observe apparent toxicity [2–4].

In summary, using human HCC data and mouse models with three approaches to inhibit Gal-1, this study unequivocally demonstrates the significant role of miR-22-Gal-1 in tumor metabolism and immune modulation, offering a compelling therapeutic target for treating HCC. Moreover, the roles of Gal-1 in various signaling pathways, such as glycosylation, complement-clotting cascades, and Rho GTPase, warrant further investigation.

## Supplementary Information

Supplementary Material 1: Cox regression analysis of miR-22 and *LGALS1* gene expression on tumor stage, age of diagnosis, and sex of the TCGA LIHC data showed hazard ratio and confidence interval, *p*-value, and proportional hazard of overall survival, disease-free interval, progression-free interval, and disease-specific survival.

## Data Availability

No datasets were generated or analysed during the current study.

## Abbreviations

AAV8: Adeno-associated virus serotype 8
AAV9: Adeno-associated virus serotype 9
HCC: Hepatocellular carcinoma
Gal-1: Galectin 1
siRNA: Small interfering RNA
miRNA: MicroRNA
AFP: α-Fetoprotein
MASH: Metabolic dysfunction-associated steatohepatitis
RA: Retinoic acid
HIF-1α: Hypoxia-inducible factor-1
DSP: Digital Spatial Profiler
TCGA: The Cancer Genome Atlas program
LIHC: Liver hepatocellular carcinoma
TCA: Tricarboxylic acid
MAC: Membrane attack complex
HDAC: Histone deacetylases
UCSC: University of California Santa Cruz
AST: Aspartate aminotransferase
ALT: Alanine transaminase
LDH: Lactate dehydrogenase
HR: Hazard ratio
PH: Proportional hazard
CI: Confidence interval
