# Supplementary material for: miR-22-Galectin-1 as an integral signaling axis in regulating metabolism and immunity in HCC
Source: Biomark Res. 2026 Jan 15;14:11. doi: 10.1186/s40364-025-00838-3 (PMC12809943; doi:10.1186/s40364-025-00838-3)
Supplement: Supplementary file 1 — Supplementary Material 1: Cox regression analysis of miR-22 and LGALS1 gene expression on tumor stage, age of diagnosis, and sex of the TCGA LIHC data showed hazard ratio and confidence interval, p-value, and proportional hazard of overall survival, disease-free interval, progression-free interval, and disease-specific survival. [file 40364_2025_838_MOESM1_ESM.docx]

**Supplementary Table 1**. Cox regression analysis of miR-22 and *LGALS1* gene expression on tumor stage, age of diagnosis, and sex of the TCGA LIHC data showed hazard ratio and confidence interval, p-value, and proportional hazard of overall survival, disease-free interval, progression-free interval, and disease-specific survival.

| **A. Overall survival** | | | | |
| --- | --- | --- | --- | --- |
| Type of survival | HR | Confidence Interval | pvalue | PH-assumption |
| Stage | 2.40 | [1.65, 3.50] | 0.0000051 | 0.23 |
| miR-22 | 0.74 | [0.61, 0.89] | 0.0018 | 0.31 |
| Age at diagnosis | 1.01 | [1.00, 1.03] | ns | 0.25 |
| LGALS1 | 1.14 | [0.96, 1.36] | ns | 0.94 |
| Sex | 0.86 | [0.58, 1.27] | ns | 0.10 |
| **B. Disease-free interval** | | | | |
| Stage | 2.30 | [1.58, 3.36] | 0.000016 | 0.18 |
| miR-22 | 0.74 | [0.62, 0.89] | 0.0014 | 0.50 |
| Sex | 1.22 | [0.83, 1.79] | ns | 0.81 |
| LGALS1 | 0.95 | [0.80, 1.12] | ns | 0.52 |
| Age at diagnosis | 1.00 | [0.99, 1.01] | ns | 0.29 |
| **C. Progression-free interval** | | | | |
| Stage | 2.12 | [1.52, 2.96] | 0.000011 | 0.56 |
| miR-22 | 0.73 | [0.62, 0.86] | 0.000129 | 0.95 |
| LGALS1 | 0.95 | [0.82, 1.10] | Ns | 0.65 |
| Age at diagnosis | 1.00 | [0.98, 1.01] | Ns | 0.32 |
| Sex | 1.00 | [0.71, 1.39] | Ns | 0.78 |
| **D. Disease-specific survival** | | | | |
| Stage | 3.52 | [2.16, 5.75] | 0.0000005 | 0.19 |
| miR-22 | 0.67 | [0.52, 0.87] | 0.00256 | 0.21 |
| LGALS1 | 1.17 | [0.93, 1.47] | ns | 0.90 |
| Sex | 0.84 | [0.50, 1.40] | ns | 0.00 |
| Age at diagnosis | 1.00 | [0.98, 1.02] | ns | 0.38 |

*HR: Hazard ratio, ns: not significant, PH: proportional hazards, LGALS1: Gal-1, ns: not significant
